# *Leinendera achaeta* sp. n., a new species of robber fly from Brazil (Diptera, Asilidae, Asilinae)

**DOI:** 10.3897/zookeys.558.6671

**Published:** 2016-02-01

**Authors:** Alexssandro Camargo, Rodrigo Vieira, Andreas Köhler, José Albertino Rafael

**Affiliations:** 1 Instituto Nacional de Pesquisas da Amazônia–INPA, CBIO–Coordenação de Pesquisas em Biodiversidade, Campus II, CEP 69080–971, Manaus, Amazonas, Brazil. Bolsista CNPq; 2 Instituto Nacional de Pesquisas da Amazônia–INPA, CBIO–Coordenação de Biodiversidade, CEP 69060–000, Manaus, Amazonas, Brazil. Bolsista FAPEAM–FIXAM; 3 Laboratório de Entomologia, Universidade de Santa Cruz do Sul–UNISC, CEP 96815–900, Santa Cruz do Sul, Rio Grande do Sul, Brazil; 4 Instituto Nacional de Pesquisas da Amazônia–INPA, CBIO–Coordenação de Pesquisas em Biodiversidade, Campus II, CEP 69080–971, Manaus, Amazonas, Brazil

**Keywords:** Asilus group, Neotropical, taxonomy

## Abstract

The third species of the Neotropical genus *Leinendera* Carrera, 1945, *Leinendera achaeta* **sp. n**., is described from Rio Grande do Sul state, Brazil. The habitus, wing and male terminalia are described and illustrated, and a key to the three Brazilian species is provided.

## Introduction

Asilinae Latreille, 1802 is the most diverse subfamily of Asilidae, including 179 extant genera, and is distributed in all biogeographic regions, except Antarctica (Geller-Grimm 2004, Londt 2005, Vieira 2012a, Artigas and Vieira 2014, Vieira and Rafael 2014). Sixty-eight genera are recognized in the Neotropical Region, of which 20 occur in Brazil (Papavero 2009, Vieira 2012a, Artigas and Vieira 2014, Vieira and Rafael 2014). Carrera (1945) had distinguished his newly proposed genus *Leinendera* through the presence of apical scutellar setae, wing with spots (of dense microtrichia) in apical third, and tergites with lateral marginal macrosetae.

**Figure 1.**
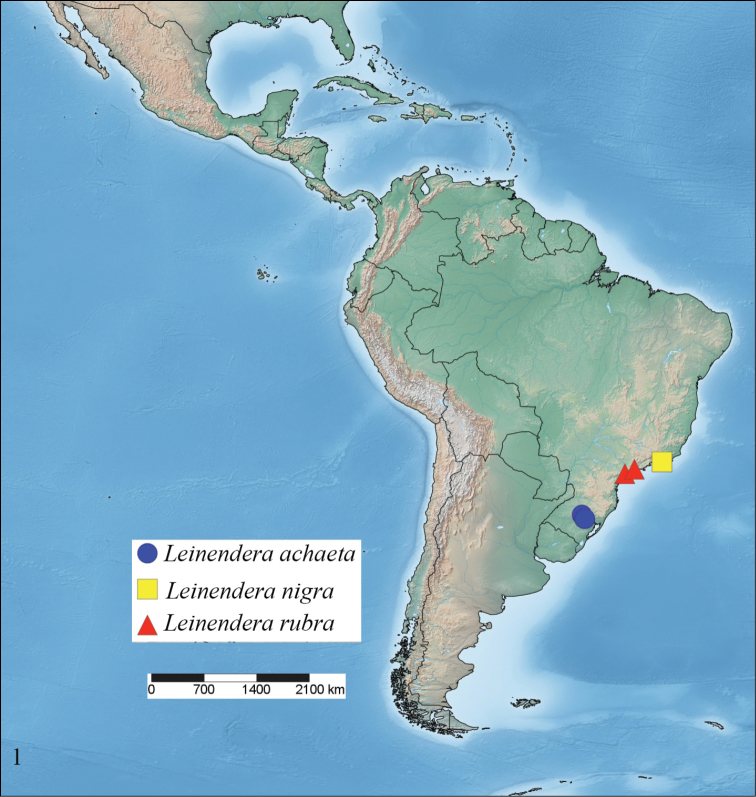
Distribution of *Leinendera* species.

Currently, the two valid species of *Leinendera* are restricted to the Neotropical Region (Fig. 1): *Leinendera rubra* Carrera, 1945 (Brazil: Rio de Janeiro and São Paulo states) and *Leinendera nigra* Vieira, 2012 (Brazil: Rio de Janeiro state) (Vieira 2012b).

In this work, the third species of *Leinendera*, from Rio Grande do Sul, Brazil, is described and illustrated, and a key to Brazilian species is provided.

## Material and methods

This study is based on the examination of specimens housed in the following institutions: CESC–Coleção Entomológica de Santa Cruz do Sul, Santa Cruz do Sul, Brazil and INPA–Instituto Nacional de Pesquisas da Amazônia, Manaus, Brazil. Morphological terminology follows Cumming and Wood (2009), and antennal terminology follows Stuckenberg (1999).

The wing was detached from the body, placed in xylene for 30 minutes and then mounted in Canada balsam between coverslips. After drying, the cover slips were glued to the edge of a piece of thick paper, which was then pinned with the specimen. The techniques of Vieira (2012b) were used to examine the terminalia. After examination and illustration, the detached parts of the terminalia were placed in microvials with glycerin and pinned with their respective specimen.

The label data are cited in full, with the original spellings, punctuation, and dates. Information presented within square brackets are complementary data not included on the labels. Data from the same specimen, but from different labels, are separated by slashes (/). The map was generated with SimpleMappr.

## Results

### Leinendera

Taxon classificationAnimaliaDipteraAsilidae

Carrera, 1945

#### Diagnosis.

Brown oblique stripe extending from the base of the wing to the base of the fore and mid coxae (Figs 2, 4, 16, 24); wing with spots (of dense microtrichia) in apical third (Figs 6, 7, 18, 26); tergites with lateral marginal macrosetae (Figs 2, 16, 24).

**Figures 2–7.**
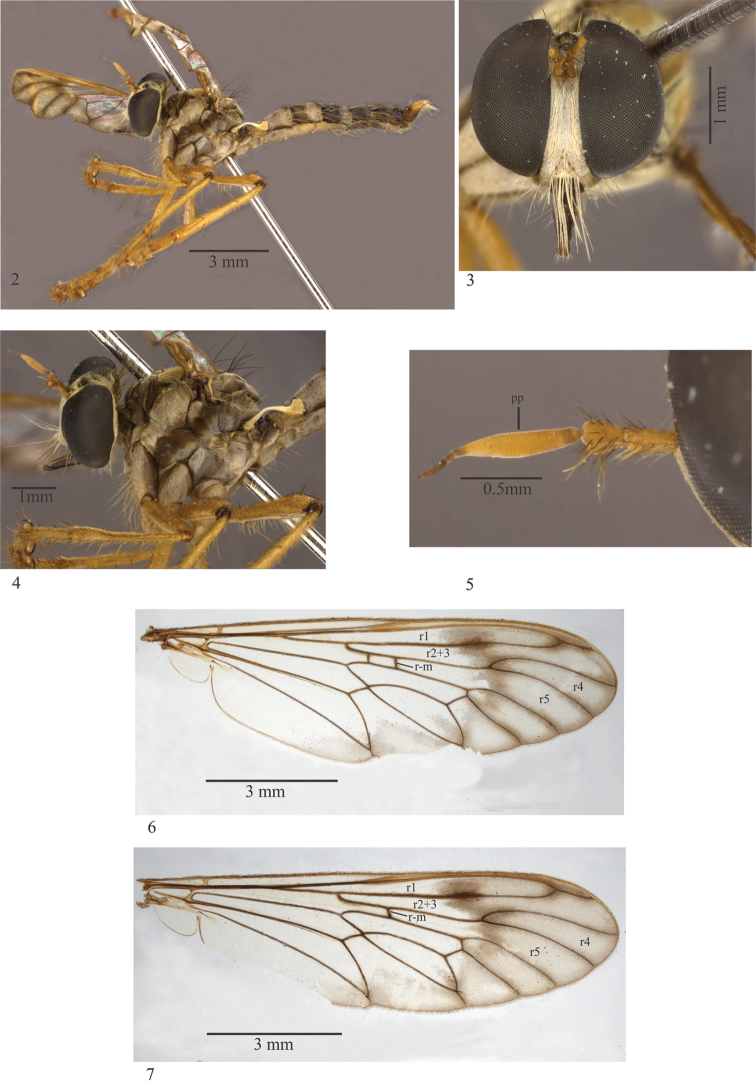
*Leinendera achaeta* sp. n. (**2–6** Holotype male.). **2** Habitus, lateral view **3** Head, frontal view **4** Head & thorax, lateral view **5** Antenna, lateral view **6** Wing **7** Paratype wing. Abbreviations: pp: postpedicel.

**Figures 8–15.**
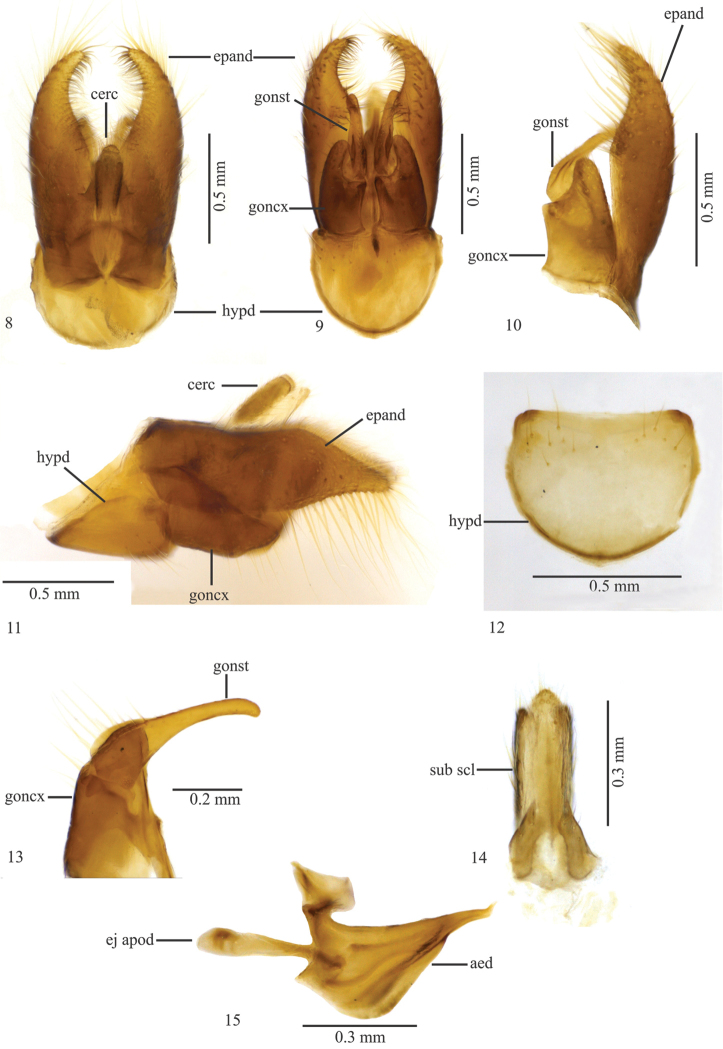
*Leinendera achaeta* sp. n. Holotype male. **8** Terminalia, dorsal view **9** Terminalia, ventral view **10** Epandrium, gonocoxite and gonostylus **11** Terminalia, lateral view **12** Hypandrium **13** Gonocoxite and gonostylus **14** Subepandrial sclerite **15** Aedeagus. Abbreviations: aed: aedeagus; cerc: cercus; ej apod: ejaculatory apodeme; epand: epandrium; goncx: gonocoxite; gonst: gonostylus; hypd: hypandrium; sub scl: subepandrial sclerite.

**Figures 16–23.**
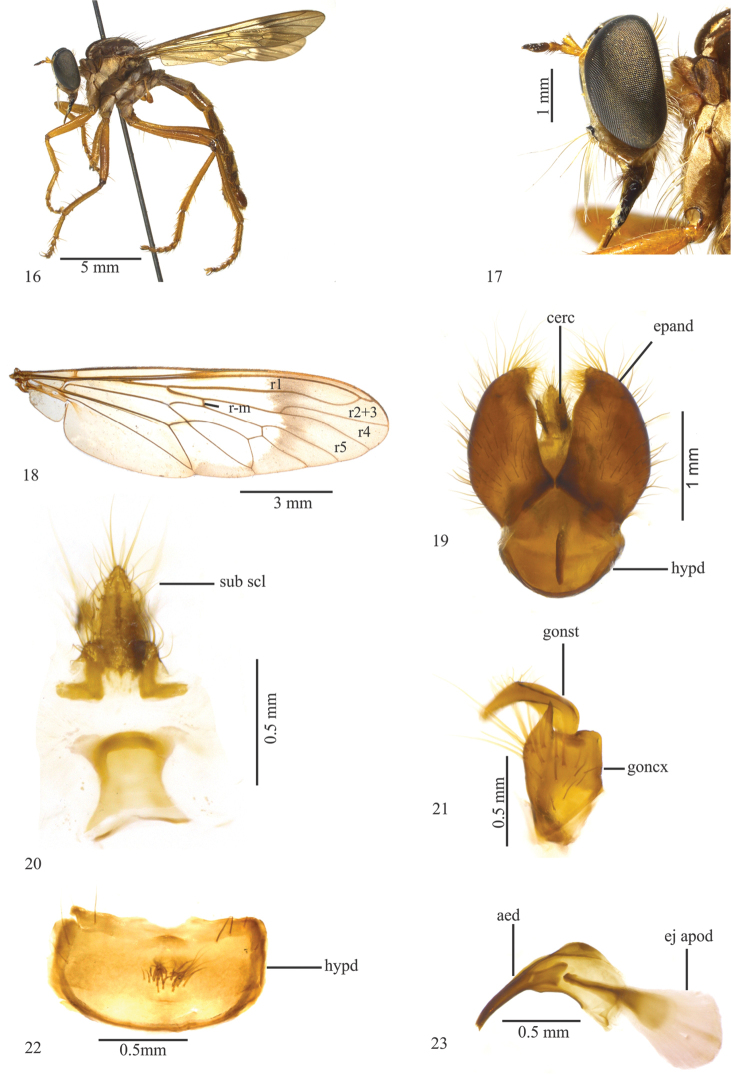
*Leinendera nigra* Vieira, 2012. Holotype male (modiﬁed from Vieira 2012). **16** Head, lateral view **17** Head, frontal view **18** Wing **19** Terminalia, dorsal view **20** Subepandrial sclerite **21** Gonocoxite and gonostylus **22** Hypandrium **23** Aedeagus. Abbreviations: aed: aedeagus; cerc: cercus; ej apod: ejaculatory apodeme; epand: epandrium; goncx: gonocoxite; gonst: gonostylus; hypd: hypandrium; sub scl: subepandrial sclerite.

**Figures 24–31.**
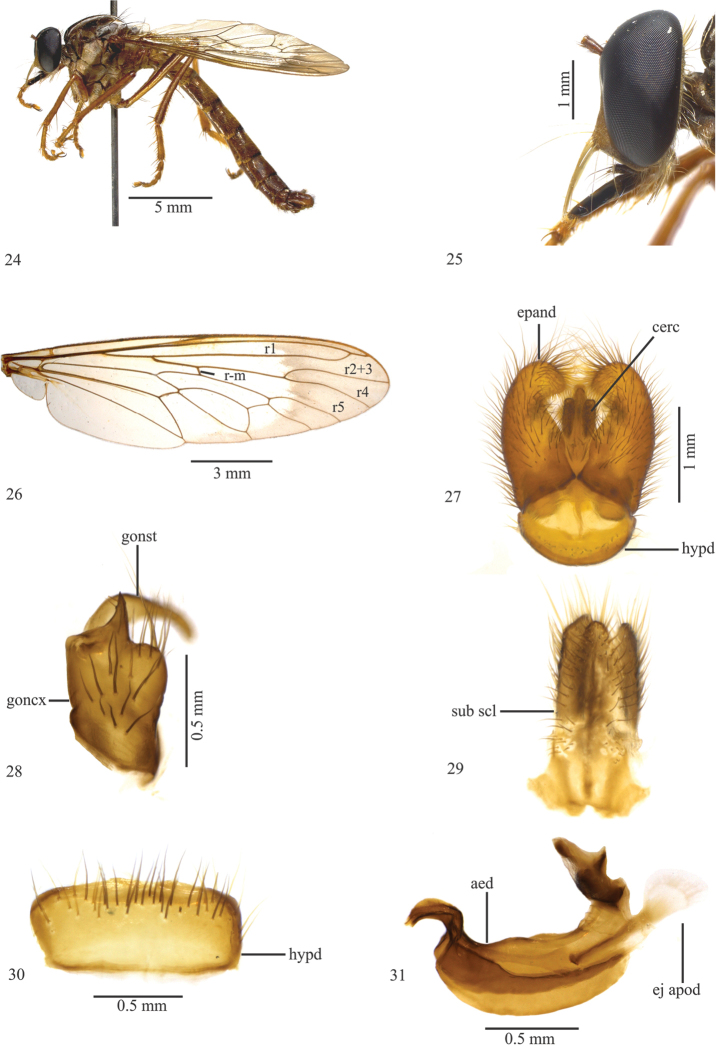
*Leinendera rubra* Carrera, 1945. Ordinary specimen male (modiﬁed from Vieira 2012). **24** Head, lateral view **25** Head, frontal view **26** Wing **27** Terminalia, dorsal view **28** Subepandrial sclerite **29** Gonocoxite and gonostylus **30** Hypandrium **31** Aedeagus. Abbreviations: aed: aedeagus; cerc: cercus; ej apod: ejaculatory apodeme; epand: epandrium; goncx: gonocoxite; gonst: gonostylus; hypd: hypandrium; sub scl: subepandrial sclerite.

### Leinendera achaeta sp. n.

Taxon classificationAnimaliaDipteraAsilidae

http://zoobank.org/D4EA37A5-B70E-4327-8ACB-EC943DAE1848

Figs 2–7 , 8–15

#### Diagnosis.

Apical scutellar macrosetae absent; epandrium elongate, apical 1/3 triangular in lateral view (Figs 8–11); gonocoxite with an indentation on apical third of the inner margin (Figs 9, 10); hypandrium with distal margin straight, basal margin rounded (Fig. 12).

**Male. Holotype.** Body: Slender (Fig. 2). Head: Antenna (Fig. 5) with yellow scape and pedicel, with brown and yellow setae; yellow postpedicel with brown apex, and 16 times the length of first element of the stylus; stylus brown; second element of stylus five times the length of the first element. Vertex (Fig. 3) golden tomentose; ocellar tubercle brown tomentose with two brown, short, proclinate, ocellar setae; face and frons golden tomentose (Fig. 3), face moderately narrow (Fig. 3), lower facial margin silvery tomentose; gena dark-brown; pale yellow mystacal macrosetae (Figs 3, 4); occiput golden-brown tomentose; yellow occipital setae; 4-6 light brown postocular macrosetae; brown palpus with brown setae; apical setae of palpus longer than others; light brown labrum, lacinia and postmentum, black labella and prementum; yellowish labial setae.

Thorax (Figs 2, 4). Antepronotum and postpronotum brown and golden tomentose; brown mesonotum; brown paramedian stripe, darker on anterior half; presutural and postsutural spots brown tomentose, area between spots grey tomentose; mesonotum grey tomentose laterally; brown scutellum with impressed rim, silvery tomentose; pleuron silvery tomentose with brown oblique stripe extending from base of wing to base of fore and mid coxae (Fig. 4). Chaetotaxy: Brown acrostichal setae; two brown notopleural macrosetae; one brown supra-alar macroseta and 1 brown supra-alar seta; one brown postalar macroseta; four brown, dorsocentral, presutural setae; four brown, dorsocentral, postsutural setae; apical scutellar macrosetae absent; one short, brown, discal scutellar seta; yellowish anatergal and katatergal setae; posterior meron + metanepisternum with yellow macroseta and pale yellow tuft of small setae on posterior margin.

Wing (Figs 6, 7). Hyaline basal 2/3, apical 1/3 extending to anal margin reaching anal cell with dense brown microtrichiae; dark brown veins; R_2+3_ slightly sinuous at the level of the R_4_ and R_5_ bifurcation; cell r4 narrower basally; without costal dilatation; R_4_ and R_5_ bifurcation beyond level of the discal cell apex; crossvein r-m before level of discal cell middle (Obs. the additional r-m on figure 6 is an anomaly); microtrichia on posterior margin arranged in two divergent planes; pale-yellow halter.

Legs (Figs 2, 4). Narrow, yellow; apex of all femora with dark brown ring. Chaetotaxy: Hind trochanter with 1 yellow macrosetae; fore femur with 4 yellow setae ventrally; mid femur with 1 yellow anterior macroseta, 2–3 yellow macrosetae anteroventrally, 2–3 yellow macrosetae posteroventrally and 1 yellow, posterior, preapical macroseta; hind femur with 2 yellow anterior macrosetae, 2–3 dorsal preapical macrosetae and 3 yellow macrosetae posteroventrally; fore tibia with 3 yellow long macrosetae laterally; mid tibia with 4 yellow long macrosetae anteroventrally, 1 yellow posterior macroseta and 2 yellow macrosetae posteroventrally; hind tibia with 3 yellow anterior setae, 2 yellow posterior setae, 1 yellow anteroventral seta and 3 yellow posteroventral setae; tarsomere with yellow setae and macrosetae; yellow empodium and pulvillus; claws with light brown basal half and black apical half.

Abdomen (Fig. 2). Brown. Dark brown tergites, except I–III light brown laterally; tergites III–VI with silvery tomentose spots laterally; tergites with yellow, lateral, marginal macrosetae. Light brown sternites, except dark brown V–VI.

Terminalia (Figs 8–15). Light brown. Epandrium elongate, apical 1/3 triangular in lateral view (Figs 8–11); hypandrium with distal margin straight, proximal margin rounded (Fig. 12); gonocoxite with an indentation on apical third of inner margin (Figs 9, 10); gonostyle tapered and with rounded apex (Fig. 13); apex of subepandrial sclerite rounded (Fig. 14); ejaculatory apodeme narrow in lateral view (Fig. 15), aedeagal sheath subtriangular in lateral view (Fig. 15).

**Length**: Body length 11.9 mm; wing length 10.9 mm.

**Holotype condition**: Right postpedicel and right hind tarsus lost. Detached wing mounted on microslides, terminalia placed in microvial with glycerin, both pinned with the specimen.

**Variation (n = 2): Size.** Body length 11.5–12.4 mm; wing length 10.1–10.5 mm. Face silvery tomentose; mid femur with 1–3 yellow anterior macrosetae; mid tibiae with 3 yellow long macrosetae anteroventrally.

**Female**: Unknown.

#### Etymology.

From the greek *achaeta*, a = absent and chaeta = bristles, referring to the absence of apical scutellar macrosetae.

#### Biology.

All specimens of *Leinendera achaeta* sp. n. were collected with Malaise traps placed in tobacco, *Nicotiana tabacum* L., plantations. The vegetation surrounding the tobacco plantations was composed mainly by grasslands and shrubs of small to medium size. No information about the prey is known.

#### Discussion.

Differs from the other two species of *Leinendera* by the absence of apical scutellar macrosetae and characters of the terminalia (Figs 2–15). When describing *Leinendera*, Carrera (1945) mentioned that the genus was distinct from *Glaphyropyga* by the presence of apical scutellar setae. However, that author described the taxon based on a single species and, with the inclusion of *Leinendera achaeta* sp. n., this character can no longer be used in the diagnosis of the genus. Regardless, *Leinendera achaeta* sp. n., *Leinendera nigra* Vieira, 2012 and *Leinendera rubra* Carrera, 1945 have a brown oblique stripe extending from the base of the wing to the base of the fore and mid coxae (Figs 16, 24), which could be used as a new diagnostic character for the genus, since it does not occur in any other closely related genus of Asilinae.

#### Type material.

Holotype: BRA[ZIL], **RS** [**Rio Grande do Sul**], Santa Cruz do Sul, Premium 08/09, 21.02.2009, Armadilha de Malaise / N: 34378 L: 6 P: H: / Holotype *Leinendera achaeta* Vieira, Camargo, Köhler & Rafael sp. nov. **(male INPA)**.

Paratypes: BRA[ZIL], **RS** [**Rio Grande do Sul**], Lagoão, 08.03.2009, Armadilha de Malaise / N: 29577 L: 108 Lote álcool: 23120 P: H: / Paratype *Leinendera achaeta* Vieira, Camargo, Köhler & Rafael **(1 male CESC)**; BRA[ZIL], **RS** [**Rio Grande do Sul**], Vera Cruz, CTA 28.11.2008, Armadilha de Malaise / N: 29657 L: 143 Lote álcool: 16444-15 P: H: / Paratype *Leinendera achaeta* Vieira, Camargo, Köhler & Rafael **(1 male CESC)**.

#### Distribution.

Brazil: Rio Grande do Sul state.

### Identification key to males of *Leinendera*

**Table:** 

1	Apical scutellar macrosetae present	**2**
–	Apical scutellar macrosetae absent	***Leinendera achaeta* sp. n.** (Brazil: Rio Grande do Sul state)
2	Lower facial margin with black projection (Fig. 17); base of r4 narrow (Fig. 18); epandrium with apex backward directed (Fig. 19); gonocoxite subquadrangular with a projection on the external margin (Fig. 20); subepandrial sclerite with a basal plate (Fig. 20); hypandrium with a tuft of short yellow setae on the middle (Fig. 22); aedeagus strongly downcurved (Fig. 23)	***Leinendera nigra* Vieira, 2012** (Brazil: Rio de Janeiro state)
–	Lower facial margin without a black projection (Fig. 25); base of r4 slightly narrow (Fig. 26); epandrium with apex inward curved (Fig. 27); gonocoxite subquadrangular with a median keel backward directed (Fig. 28); subepandrial sclerite simple, without projections (Fig. 29); hypandrium with setae arranged along entire posterior margin (Fig. 30); aedeagus upcurved (Fig. 31)	***Leinendera rubra* Carrera, 1945** (Brazil: Rio de Janeiro and São Paulo states)

## Supplementary Material

XML Treatment for Leinendera

XML Treatment for Leinendera achaeta
